# The potential of integrating human and mouse discovery platforms to advance our understanding of cardiometabolic diseases

**DOI:** 10.7554/eLife.86139

**Published:** 2023-03-31

**Authors:** Aaron W Jurrjens, Marcus M Seldin, Corey Giles, Peter J Meikle, Brian G Drew, Anna C Calkin

**Affiliations:** 1 Baker Heart and Diabetes Institute Melbourne Australia; 2 Central Clinical School, Monash University Melbourne Australia; 3 Department of Biological Chemistry and Center for Epigenetics and Metabolism, University of California, Irvine Irvine United States; 4 Baker Department of Cardiometabolic Health, University of Melbourne Melbourne Australia; 5 Baker Department of Cardiovascular Research Translation and Implementation, La Trobe University Bundoora Australia; University of Sydney Australia; University of Sydney Australia

**Keywords:** systems genetics, multi-omics, genetic reference panels, genome-wide association studies, genetic mapping, Hybrid Mouse Diversity Panel, cardiometabolic disease, non-alcoholic fatty liver disease, atherosclerosis, coronary artery disease

## Abstract

Cardiometabolic diseases encompass a range of interrelated conditions that arise from underlying metabolic perturbations precipitated by genetic, environmental, and lifestyle factors. While obesity, dyslipidaemia, smoking, and insulin resistance are major risk factors for cardiometabolic diseases, individuals still present in the absence of such traditional risk factors, making it difficult to determine those at greatest risk of disease. Thus, it is crucial to elucidate the genetic, environmental, and molecular underpinnings to better understand, diagnose, and treat cardiometabolic diseases. Much of this information can be garnered using systems genetics, which takes population-based approaches to investigate how genetic variance contributes to complex traits. Despite the important advances made by human genome-wide association studies (GWAS) in this space, corroboration of these findings has been hampered by limitations including the inability to control environmental influence, limited access to pertinent metabolic tissues, and often, poor classification of diseases or phenotypes. A complementary approach to human GWAS is the utilisation of model systems such as genetically diverse mouse panels to study natural genetic and phenotypic variation in a controlled environment. Here, we review mouse genetic reference panels and the opportunities they provide for the study of cardiometabolic diseases and related traits. We discuss how the post-GWAS era has prompted a shift in focus from discovery of novel genetic variants to understanding gene function. Finally, we highlight key advantages and challenges of integrating complementary genetic and multi-omics data from human and mouse populations to advance biological discovery.

## Introduction

Cardiometabolic diseases encompass a range of interrelated conditions that develop from underlying metabolic perturbations precipitated by genetic, environmental, and lifestyle factors. A significant burden of disease in developed countries is attributable to these conditions including coronary artery disease (CAD), heart failure (HF), obesity, type 2 diabetes (T2D), and non-alcoholic fatty liver disease (NAFLD) (Tsao et al., 2022; Younossi et al., 2018). Traditional risk factors that increase an individual’s risk of developing cardiometabolic diseases include obesity, dyslipidaemia, smoking, and insulin resistance. However, cardiometabolic diseases can manifest in the absence of traditional risk factors, highlighting a gap in our understanding of the causative factors that drive disease initiation and progression. The variable presentation of cardiometabolic diseases is largely due to the complex interaction of genetic and environmental factors on subsequent downstream biological pathways. Specifically, genetics is estimated to explain 40–60% of CAD, 20–70% of NAFLD, and 26% of HF (Sookoian and Pirola, 2017; McPherson and Tybjaerg-Hansen, 2016; Lindgren et al., 2018). Indeed, a preponderance of loci have been linked with cardiometabolic diseases (Mahajan et al., 2018; Roselli et al., 2018; Koyama et al., 2020; Erdmann et al., 2018; Fairfield et al., 2022; Atanasovska et al., 2015), demonstrating the polygenic nature of these conditions. Importantly, many of the genetic mechanisms that drive cardiometabolic diseases are conserved across mammalian species (Li et al., 2020; Talukdar et al., 2016; Koplev et al., 2022; von Scheidt et al., 2017; Benegiamo et al., 2023). Thus, model organisms provide an alternate and often complementary approach for exploring novel mechanisms that underlie cardiometabolic diseases. Furthermore, model organisms can lend themselves to complex and/or invasive phenotyping (e.g. tissue collection/biopsy or magnetic resonance imaging) and interventions (e.g. genetic manipulation or trial drug screens) that cannot readily be undertaken in humans. They can also be studied across multiple generations, with tight environmental control, and pertinent tissue samples are readily available for molecular analyses.

In this review, we provide an overview of mouse genetic reference panels and the opportunities they provide for the study of cardiometabolic diseases and relevant traits, with particular emphasis on the Hybrid Mouse Diversity Panel (HMDP). We discuss how systems genetics research has shifted focus in the post-genome-wide assocation study (GWAS) era from discovery of novel genetic variants, to understanding gene function. In doing so, we highlight the substantial contributions that systems geneticists have made to advance the field (for comprehensive reviews, readers are directed to Baliga et al., 2017; Civelek and Lusis, 2014; Li and Auwerx, 2020; Seldin et al., 2019). Furthermore, we expand upon previous contributions, by discussing some key advantages and challenges of integrating complementary genetic and multi-omics data from human and mouse populations to reveal novel biological insight, as touched on previously by others (Ashbrook and Lu, 2021; Li and Auwerx, 2020; Nadeau and Auwerx, 2019; Votava and Parks, 2021). We conclude with a discussion of future considerations for the field.

### Systems genetics

Systems genetics is an integrative, population-based methodology that explores the relationship between genetics and phenotypes, with the goal of understanding how genetic variance impacts complex traits. Mechanistic links are prioritised by investigating how the abundance of intermediate phenotypes, such as RNA, proteins, lipids, or other metabolites, co-operate to precipitate complex traits. As such, genotype serves as a causal anchor to guide analyses of transcriptomics, proteomics, metabolomics, phenomics, and other omics data to associate with complex traits. By integrating various layers of omics data, we can construct a comprehensive representation of complex biological networks in a given cell, tissue, individual, or population, facilitating discovery of novel molecular targets. These approaches, combined with the continued development of advanced statistical and algorithmic modelling, enables unprecedented predictive capabilities.

### Genome-wide association studies

Over 20 years ago, the Human Genome Project generated the first detailed annotation of the ~3 billion base pair human DNA sequence (Venter et al., 2001). This seminal advance paved the way for researchers to leverage genetic information between individuals, to infer causality in phenotype and disease outcomes. Thereafter, GWAS were spawned, enabling a paradigm shift in our approach to biological discovery. In essence, GWAS involves the mapping of single nucleotide polymorphisms (SNPs) across the entire genome and identifying their association with a given trait. A locus that maps to a specific trait is known as a quantitative trait locus (QTL), where one or more potentially causal SNPs typically reside. Such traits include, but are not limited to, mRNA expression (eQTL), epigenetic markers such as DNA methylation (meQTL), the abundance of a protein (pQTL), lipid (lQTL) or metabolite (mQTL), or a disease phenotype (pheQTL). Further, eQTLs and pQTLs can be distinguished by the genomic coordinates of the gene or protein of interest, where QTLs that associate locally (~1–10 Mb) to the encoded region are referred to as *cis* (i.e. acting via contiguous genomic structures), while distal associations are referred to as *trans* (i.e. acting via distant genomic architecture). In the context of germline associations to molecular traits such as expression, DNA plays a regulatory role in the production of RNA and consequent protein, and therefore, co-mapping of a *cis*-eQTL with a given trait infers a potential causal relationship, although experimental validation is usually required for confirmation of such findings.

Human GWAS have uncovered many genes that contribute to complex diseases including NAFLD (Anstee et al., 2020), CAD (Deloukas et al., 2013; Nikpay et al., 2015), and obesity (Pulit et al., 2019). However, despite the potential of this approach, in practice, most GWAS for complex traits observe associations for common variants with modest effect sizes. Further, failure of loci to be replicated in subsequent studies further complicates interpretation (van der Laan et al., 2015; Siontis et al., 2010). This is thought to be due, in part, to the large environmental variability, underpowered study cohorts, complex genetic aetiology, and differences across ethnicities, where allele frequencies often differ, and where many population-specific SNPs are not present in most standard genotyping arrays. Furthermore, a major proportion of genome-wide association (GWA) loci for complex traits reside in non-coding DNA and can exert effects on distant, though related gene networks (Erdmann et al., 2018; Li et al., 2016). Thus, unravelling the causal mechanisms for robust associations can prove to be extremely challenging.

It is becoming increasingly evident that the major proportion of complex conditions, including cardiometabolic diseases, are influenced by hundreds to thousands of subtle genetic variants in combination (Boyle et al., 2017; Ritchie et al., 2021), indicating their polygenic nature, as opposed to being driven by a single genetic variant (monogenic). A complementary approach to quantify the genetic contribution to disease risk is to generate a polygenic score (PGS), which aggregates the influence of multiple variants to predict an individual’s genetic predisposition for a particular trait or clinically diagnosed disease, including cardiometabolic diseases such as CAD and NAFLD (Inouye et al., 2018; Sun et al., 2021; Namjou et al., 2019). Since PGS are able to capture risk that is often independent of, and thus complementary to, traditional risk factors, they can facilitate additional clinical risk stratification, as has been recently reviewed (Polygenic Risk Score Task Force of the International Common Disease Alliance, 2021). By combining PGS with multi-omics data, such approaches can provide an investigative resource for the identification of novel diagnostic and therapeutic targets (Ritchie et al., 2021).

### Human genetic and multi-omics resources

Several large-scale human population studies have been undertaken to generate resources comprising clinical, molecular, genetic, and omics-derived data. A non-exhaustive list of relevant resources is provided in Table 1. Some studies serve as a resource for parallel analysis of genotype or multi-tissue gene expression with a compendium of clinical and molecular traits, such as the UK Biobank or the Genotype-Tissue Expression project (GTEx); others are useful for the study of more specific traits, such as the Myocardial Applied Genomics Network (MAGNet) for the study of HF, the Stockholm-Tartu Atherosclerosis Reverse Networks Engineering Task (STARNET) for the study of atherosclerosis, and the METabolic Syndrome In Man (METSIM) for metabolic phenotypes. Such resources are cumulative and improve in power as sample size increases. Biological samples can be stored and later analysed to complement previous datasets and provide additional biological insight. For example, integration of recent lipidomic analysis of historical plasma samples from ~4500 individuals from the Busselton Health Study (BHS) identified many loci associated with CAD susceptibility that co-localised with lipid loci, suggesting shared genetic aetiology (Cadby et al., 2020; Cadby et al., 2022).

**Table 1.** Human genetic and multi-omics resources for cardiometabolic traits.

Resource	Population	Tissue(s)	*Genetic and omics data	*Primary phenotypes	Link
**METSIM** METabolic Syndrome In Men Study	n=10,197 Finnish males, aged 45–73	Subcutaneous adipose tissue	Exome array genotyping (Huyghe et al., 2013) WGS (Yin et al., 2022; Ganel et al., 2021) WES (Locke et al., 2019) Adipose tissue DNA methylation (Orozco et al., 2018) Adipose tissue transcriptomics (Gusev et al., 2016) Plasma metabolomics (Yin et al., 2022)	Medical history Clinical and metabolic traits Cardiovascular disease risk factors Medication usage Oral glucose tolerance test (Stancáková et al., 2009)	Reviewed in Laakso et al., 2017
**MAGNet** Myocardial Applied Genomics Network	n=177 cases and n=136 controls for heart failure; collected during transplant	Cardiac tissue	DNA genotyping (Lin et al., 2014) WGS CHIP-Seq (Tan et al., 2020) Single-nuclear RNA-Seq (Tucker et al., 2020) ATAC-Seq Cardiac transcriptomics (Roselli et al., 2018; Lin et al., 2014; Liu et al., 2015)	Cardiomyopathy classification	https://www.med.upenn.edu/magnet/
**STARNET** Stockholm-Tartu Atherosclerosis Reverse Networks Engineering Task study	n=600 cases and n=250 controls for CAD; individuals undergoing open-thoracic surgery	Aortic root, mammary artery, liver, subcutaneous fat, visceral fat, skeletal muscle, whole blood	DNA genotyping (Hägg et al., 2009) Transcriptomics (Hägg et al., 2009; Franzén et al., 2016)	Clinical and biomedical traits Medical history Medication usage Preoperative angiographic assessment of CAD History of CAD and stroke	http://starnet.mssm.edu/
**GTEx** Genotype-Tissue Expression project	n=948 donors, aged 21–70; Biospecimens collected <24 hr post-mortem	54 tissue types	DNA genotyping (GTEx Consortium, 2015) WGS (GTEx Consortium, 2020) WES Transcriptomics (GTEx Consortium, 2015; GTEx Consortium, 2020) Proteomics (29 tissues) (Jiang et al., 2020)	Medical history Disease risk factors Cause of death	https://gtexportal.org/
**UKB** UK Biobank	~500,000 individuals of European descent from the UK; with longitudinal follow-up on some subsets	Blood, urine, and saliva samples	DNA genotyping (Bycroft et al., 2018) WGS (Halldorsson et al., 2022) WES (Van Hout et al., 2020; Szustakowski et al., 2021) Metabolomics (Julkunen et al., 2021) Plasma proteomics (Sun et al., 2022)	Medical history Health records Clinical biomarkers Physical activity monitors (Khurshid et al., 2021) Online questionnaires Several imaging modalities (Littlejohns et al., 2020) Psychosocial factors and environmental exposures (Mutz et al., 2021)	https://www.ukbiobank.ac.uk/
**CARDIoGRAMplusC4D** Coronary ARtery DIsease Genome-wide Replication and Meta-analysis plus The Coronary Artery Disease study	n=63,746 cases and n=130,681 controls for CAD or MI	-	-	Meta-analysis of numerous published and unpublished GWAS in individuals with European ancestry Case/control status for CAD and MI (Deloukas et al., 2013; Preuss et al., 2010; Schunkert et al., 2011; The Coronary Artery Disease (C4D) Genetics Consortium, 2011)	http://www.cardiogramplusc4d.org/
**BHS** Busselton Health Study	>5000 individuals from Busselton, Western Australia	Plasma	DNA genotyping (Cadby et al., 2018) Lipidomics (Cadby et al., 2020; Cadby et al., 2022)	Clinical and biomedical traits Self-reported medical history	https://bpmri.org.au/research/key-projects-studies/busselton-health-study-2.html
**MVP** Million Veterans Project	n>900,000 veterans from the United States, aged 50–69	Blood	DNA Genotyping (Giri et al., 2019) WGS WES	Self-reported medical history Electronic health records	https://www.mvp.va.gov

* For brevity, a subset of relevant datatypes and key references are provided in this table. We apologise to the investigators whose work could not be cited due to space limitations. See accompanying links and references for additional information.

WGS, whole genome sequencing; WES, whole exome sequencing; SNP, single nucleotide polymorphism; CAD, coronary artery disease; MI, myocardial infarction; CHIP-seq, Chromatin Immunoprecipitation Sequencing; ATAC-Seq, assay for transposase-accessible chromatin using sequencing; RNA-Seq, RNA sequencing; GWAS, genome-wide association study.

Dissection of the genetic component of disease is, however, further complicated by the variable influence of environmental factors on differing genetic backgrounds, known as gene-by-environment (G×E) interactions. Even traits that are highly heritable and penetrable, such as obesity, are amenable to environmental influence and indeed genetic background (Brandkvist et al., 2020; Abadi et al., 2017), which has historically been difficult to control in humans, though efforts to collect extensive data on lifestyle and environmental factors are now emerging in the UK Biobank (Mutz et al., 2021). These challenges have prompted researchers to search for alternative approaches to minimise environmental influences to more accurately estimate the genetic component of a given disease.

### Mouse genetic reference panels

Studying genetically diverse reference populations is a complementary approach to analysing human genetics, particularly as environment can be tightly controlled, and genetic background can be replicated across cohorts and conditions. Genetic reference panels (GRPs) of model organisms including worms (*Caenorhabditis elegans*) (Cook et al., 2017), flies (*Drosophila melanogaster*) (Mackay et al., 2012), mice (Bennett et al., 2010), and rats (Tabakoff et al., 2019) are a cornerstone of systems genetics research and over the past several decades, have yielded considerable biological insight, underscoring the potential of these approaches to expand our understanding of how genetics influence biological traits. A key consideration regarding the use of GRPs is the appropriate selection of a model organism that approximates human health and/or disease (Li et al., 2020; von Scheidt et al., 2017; Li et al., 2019). Panels of genetically diverse mice have been particularly useful in the study of cardiometabolic diseases and related traits as they possess many biological similarities to humans. In this context, valuable resources for linking conserved gene-trait associations with underlying biological mechanisms have been provided by mouse GRPs such as the BXD (C57BL/6J × DBA/2J) lines (Ashbrook et al., 2021), Collaborative Cross (CC) (Churchill et al., 2004), Diversity Outbred (DO) (Churchill et al., 2012), Um-Het3 (Het3) (Miller and Chrisp, 1999), ILSXISS (Williams et al., 2004), and HMDP (Bennett et al., 2010). Genetically diverse mouse platforms overcome many of the limitations previously identified with human GWAS. Notably, tissue samples are readily obtainable for molecular and cellular analyses, while external sources of variation such as diet, co-morbidities, and environmental conditions can be tightly controlled. Reducing external sources of variation increases confidence in the identification of phenotypic variation that is attributable to genetics. Notably, the properties of the various mouse GRPs, as we will discuss, have implications for their application. For example, inbred mice are deliberately bred to homozygosity at each genetic locus, allowing mice with isogenic backgrounds to be tested under multiple environmental conditions and thus, relative genetic, environmental, or G×E quantified directly. On the other hand, outbred mice are heterozygous at most loci and better represent the genetic architecture of humans and therefore, in some instances, may be advantageous compared to other panels when mapping highly polygenic traits (Keele, 2023). Alternatively, inbred mice can be intercrossed to generate F_1_ hybrid progeny to combine the advantages of both inbred and outbred panels – reproducibility and heterozygosity, albeit with more breeding complexity (Ashbrook et al., 2021; Keele, 2023; Threadgill et al., 2002). This breeding strategy can also be leveraged to cross genetically engineered lines (i.e. transgenic models) onto a diverse genetic background to confer disease susceptibility (Bennett et al., 2015; Neuner et al., 2019a; Neuner et al., 2019b). Importantly, the total genetic diversity across strains (i.e. ~71 million segregating SNPs across 36 inbred mouse strains Doran et al., 2016) is comparable to that which might be observed in a human population (i.e. ~84.7 million SNPs across 26 human populations Auton et al., 2015).

With the ongoing development of high-throughput sequencing technologies, mouse genomes can be readily sequenced at high fidelity, and through integration with complementary omics data, regulatory loci can be identified with high confidence. Whole genome sequencing is available for all BXD lines and many CC strains, while SNP array data is available for most inbred mouse panels (Bennett et al., 2010; Ashbrook et al., 2021; Shorter et al., 2019; Yang et al., 2009). Since the genetics of inbred mice remain mostly stable over subsequent generations, with the exception of rare spontaneous mutations (Ashbrook et al., 2020), genotyping is usually not required for successive studies. Mouse GRPs can also be subjected to specific environmental ‘perturbations’ such as diet (Parks et al., 2013), drug treatments (Gatti et al., 2018; Harrill et al., 2009; Pirie et al., 2019), exercise interventions (Moore et al., 2019), or pathological insults that mimic human disease settings (Wang et al., 2016). Phenotyping undertaken in the absence or presence of such perturbations can be especially useful in the study of complex diseases sensitive to environmental influence. Meta-analysis of multiple GRPs can improve association mapping power and resolution and capture G×E interactions (Kang et al., 2014; Furlotte et al., 2012).

Another application of mouse GRPs is the generation of a mouse cellular GRP, made up of cultured primary cells derived from GRP strains, which can be leveraged as an *ex vivo* genetic screen (reviewed in Swanzey et al., 2021). Cultured cells isolated from many strains of a GRP can be studied in response to specific treatments to provide detailed insights into gene-by-gene (G×G) or G×E interactions. For example, cultured islets from ~500 DO mice that were maintained on a high-fat, high-sucrose (HF/HS) diet for ~22 weeks (Keller et al., 2018) were subsequently investigated for their response to insulin secretagogues *ex vivo* (Keller et al., 2019). This facilitated the identification of genes involved in the regulation of insulin secretion, several of which were later validated in transgenic mouse models. A similar approach has been used to identify novel regulators of the insulin secretory response in pancreatic islets from BXD mice (Berdous et al., 2020) and the acute response to inflammatory stimuli in primary macrophages from HMDP mice (Orozco et al., 2012).

### Current mouse GRPs

Due to the differences in founder strain selection and breeding strategies, the various mouse GRPs can differ in several aspects including the number of strains, magnitude of genetic diversity, mapping power, mapping resolution, and strain reproducibility. Of note, parameters such as the power and resolution of association mapping are dependent on factors such as number of strains, replicates per strain, and depth of sequencing. Such considerations have been discussed for panels consisting of inbred, outbred, and F_1_ hybrid mice (Keele, 2023). To date, a comprehensive comparison of relevant factors such as association mapping power and resolution has yet to be undertaken across all mouse GRPs, but would offer insightful comparisons when considering which panel is most suitable for a given trait or study. An overview of common mouse GRPs and their application for the study of cardiometabolic diseases is provided in Table 2. These populations have been subjected to a variety of conditions across many common diseases and have been extensively reviewed elsewhere (Ashbrook et al., 2021; Churchill et al., 2012; Collaborative Cross Consortium, 2012; Lusis et al., 2016), whereas this review will focus on the HMDP, in particular.

**Table 2.** Common mouse genetic reference panels utilised for the study of cardiometabolic diseases.

Breeding structure	Panel	Description	Strains	Advantages	Constraints	*Application of panels for cardiometabolic-related phenotypes
*Inbred*	**BXD** C57BL/6J × DBA/2J	Inbred mouse panel derived from intercrosses of C57BL/6J and DBA/2J strains (Ashbrook et al., 2021) http://www.genenetwork.org	198 strains derived from: C57BL/6J, DBA/2J	Most inbred strains are readily available (i.e. JAX labs) Data available for several thousand phenotypes and >100 omics datasets Large quantity of strains enables enormous mapping power	Lower mapping precision and genetic diversity compared to multi-parent populations Less genetic diversity than outbred mice due to homozygosity at each loci	Lipid metabolism (Jha et al., 2018b; Jha et al., 2018a) Body weight (Roy et al., 2021) Atherosclerosis (Colinayo et al., 2003) Blood pressure (Koutnikova et al., 2009) NAFLD progression (Zhu et al., 2020) Cardiac hypertrophy/pathology (Chen et al., 2020) Liver mitochondrial function (Williams et al., 2016)
	**CC** Collaborative Cross	Inbred mouse panel derived from intercrosses between eight progenitor strains (Collaborative Cross Consortium, 2012)	~100 strains derived from: A/J, C57BL/6J, 129S1/SvImJ, NOD/ShiLtJ, NZO/H1LtJ, CAST/EiJ, PWK/PhJ, WSB/EiJ	Captures a relatively large proportion of the genetic diversity in mice due to being a multi-parent population High genetic diversity; ~45 million segregating SNPs Strains fully genotyped	Less genetic diversity than outbred mice due to homozygosity at each locus	Body weight (Yam et al., 2022) NASH/NAFLD (Abu-Toamih Atamni et al., 2016a; de Conti et al., 2020) Diabetes/IR (Abu-Toamih Atamni et al., 2016b; Abu-Toamih Atamni et al., 2017; Abu-Toamih Atamni et al., 2019) Cardiac pathology (Zeiss et al., 2019) Response to exercise (McMullan et al., 2018; Mathes et al., 2011)
	**HMDP** Hybrid Mouse Diversity Panel	Diverse mouse panel derived from intercrosses of classical and recombinant inbred strains (Lusis et al., 2016) http://www.genenetwork.org	>130 strains derived from: C57BL/6J, DBA/2J, A/J, C3H/J, BALBc/J	Captures a relatively large proportion of the genetic diversity in mice due to multi-parent ancestry and inclusion of wild-derived strains	Less genetic diversity than outbred mice due to homozygosity at each loci	Lipid metabolism (Bennett et al., 2010; Parker et al., 2019; Norheim et al., 2021) Body weight (Parks et al., 2013) NASH/NAFLD (Hui et al., 2018; Hui et al., 2015; Chella Krishnan et al., 2018; Norheim et al., 2021; Kurt et al., 2018; Norheim et al., 2017; Chella Krishnan et al., 2021a) Diabetes/IR (Parks et al., 2015; Norheim et al., 2018) Atherosclerosis (Talukdar et al., 2016; Koplev et al., 2022; von Scheidt et al., 2017; Bennett et al., 2015; Cohain et al., 2021; Kessler et al., 2017) Cardiac hypertrophy/pathology (Wang et al., 2016; Rau et al., 2015; Santolini et al., 2018; Rau et al., 2017; Wang et al., 2019; Seldin et al., 2017; Lin et al., 2018; Cao et al., 2022; Krishnan et al., 2022) Exercise metabolism (Moore et al., 2019) Multiple cardiometabolic-related traits (Civelek et al., 2017; Chella Krishnan et al., 2019; Chella Krishnan et al., 2021b; Norheim et al., 2019; Seldin et al., 2018)
	**ILSXISS**	Diverse panel of recombinant inbred mice derived from ILS and ISS progenitor strains (DeFries et al., 1989) http://www.genenetwork.org	~77 strains derived from: ILS, ISS; both of which are in turn derived from: A, AKR, BALB/c, C3H/2, C57BL, DBA/2, Is/Bi and RIII	Alternative inbred cross that provides differing foundational strains and therefore diversity.	Less genetic diversity than outbred mice due to homozygosity at each loci	Body weight/adiposity (Liao et al., 2011; Rikke et al., 2006; Bennett et al., 2005) Metabolic response to dietary challenge (Mulvey et al., 2021; Yau et al., 2021) IR (Stöckli et al., 2017; Nelson et al., 2022)
*F*_*2*_ *Hybrid*	**Het3** Um-Het3	Heterogenous mouse population mostly used in ageing research (Nadon et al., 2008) https://www.nia.nih.gov/research/dab/interventions-testing-program-itp	Able to generate unlimited genetically distinct mice, derived from a four-way cross between (BALB/cJ × C57BL6/J) F_1_ females with (C3H/HeJ × DBA/2J) F_1_ males	F_2_ offspring are derived from parents with known linkage phase, allowing for the study of parent-of-origin effects Each mouse is genetically unique High allelic variation between F_2_ offspring The population is reproducible, allowing comparison of genetic and phenotypic data across generations, provided sample sizes are sufficiently large	Genotyping is required for each mouse for genetic association studies	Compounds with effects on cardiometabolic-related traits (Zhu et al., 2022; Miller et al., 2014; Herrera et al., 2020; Miller et al., 2020; Snyder et al., 2023) Impact of dietary conditions on cardiometabolic-related traits (Miller et al., 2014; Zheng et al., 2022; Green et al., 2022)
*Outbred*	**DO** Diversity Outbred	Stocks of genetically unique outbred mice derived from eight CC progenitor strains (Churchill et al., 2012)	Able to generate unlimited genetically distinct stocks of mice, derived from: A/J, C57BL/6J, 129S1/SvImJ, NOD/ShiLtJ, NZO/H1LtJ, CAST/EiJ, PWK/PhJ, WSB/EiJ	High mapping precision due to extensive allelic diversity Captures ~90% of the genetic diversity in laboratory mice	Requires more mice to achieve comparable statistical power to inbred designs Cannot measure intra-strain response to intervention Each mouse requires genotyping for GWA analysis	Lipid metabolism (Coffey et al., 2017; Linke et al., 2020) Body weight (Wright et al., 2022) Atherosclerosis (Smallwood et al., 2014) Diabetes/IR (Keller et al., 2018; Keller et al., 2019) Cardiac hypertrophy (Starcher et al., 2021) Hepatic mRNA and miRNA expression (Coffey et al., 2017; Que et al., 2021) Multiple cardiometabolic-related traits (Svenson et al., 2012; Tyler et al., 2017)

* For brevity, a selection of key phenotypes and references are provided in this table. We apologise to the investigators whose work could not be cited due to space limitations.

NAFLD, non-alcoholic fatty liver disease; NASH, non-alcoholic steatohepatitis; IR, insulin resistance; SNP, single nucleotide polymorphism; GWA, genome-wide association;.

### BXD

The BXD panel consists of 198 strains of inbred mice derived from C57BL/6J and DBA/2J parental strains and was initially used to map Mendelian traits upon its inception in the 1970s (Ashbrook et al., 2021; Taylor et al., 1973; Taylor et al., 1999). BXD mice have been studied for phenotypes relating to lipid metabolism (Jha et al., 2018b; Jha et al., 2018a), atherosclerosis (Colinayo et al., 2003), blood pressure (Koutnikova et al., 2009), NAFLD progression (Zhu et al., 2020), and HF (Chen et al., 2020), among others. There are many advantages to using the BXD panel to map complex traits. Firstly, BXD mice provide sufficient statistical power for association mapping using limited numbers of mice due to homozygosity at each locus, increased relative allele frequencies throughout the population (given that the panel is derived from two founder strains), and fully sequenced genomes (Ashbrook et al., 2021). Secondly, using updated SNP markers, robustly detectable traits can be mapped with sufficient resolution using only 60–80 strains, although the inclusion of more strains can obviously improve mapping resolution further (Ashbrook et al., 2021). In general, increasing the number of replicate animals within each strain improves statistical power, while increasing the number of genetically distinct strains refines resolution. Thirdly, the ability to reproduce isogenic strains for replication across studies has facilitated the accumulation of several thousand classical phenotypes with >100 omics datasets over the past 50 years (Ashbrook et al., 2021), accessible on GeneNetwork.org. One recognised limitation of the BXD panel is that it captures a lower proportion of the total genetic diversity in mice in comparison to more diverse multi-parent populations such as the HMDP, CC, or the DO (Roberts et al., 2007). Hence, this platform is confined in its capacity to capture polymorphisms that are not represented in the two founder strains and may translate to comparatively reduced phenotypic variation for some traits (Philip et al., 2011).

### Collaborative Cross (CC)

The Complex Trait Consortium developed three genetically diverse mouse GRPs in the early 2000s in an effort to advance systems genetics (Churchill et al., 2004). Firstly, the eight recombinant inbred founder strains, made up of five common laboratory strains and three wild-derived inbred strains, capture a significant amount of the genetic diversity observed in mice (Roberts et al., 2007). Secondly, the founder strains were subsequently combined and inbred to generate ~100 genetically stable, recombinant inbred lines which make up the CC (Collaborative Cross Consortium, 2012; Noll et al., 2019). Due to a low survival rate and infertility, the CC was unable to be expanded to 1000 inbred strains as was initially projected. Despite this, the CC is well suited to capture a substantial proportion of the genetic variation that exists in mice, where replication is possible (Keele et al., 2019). One additional consideration with the limited number of strains available is that association mapping resolution and power remains relatively limited, especially for less frequent alleles within the cross. This panel has been used to map many complex traits related to body composition, exercise physiology, energy metabolism, and behavioural traits (Philip et al., 2011; McMullan et al., 2018; Mathes et al., 2011), and inspired the generation of the DO panel.

### Diversity Outbred (DO)

The DO is a resource of non-reproducible, genetically unique stocks of mice generated by randomised outbreeding of incipient CC strains during the early stages of inbreeding (Churchill et al., 2012). A major advantage of the DO is the extensive diversity of allelic combinations compared with inbred panels, which can be leveraged to identify susceptible loci with higher resolution mapping capacity, with a large number of mice (Logan et al., 2013). However, the high allelic diversity and limited pre-definition of linkage structure also comes with more noise. Consequently, significantly more mice are required to achieve the same statistical differences using association mapping (linear mixed models and ‘logarithm of the odds’ scoring) as inbred panels. Studies using the DO have yielded insight into the genetic architecture of cardiometabolic diseases and related traits such as atherosclerosis, plasma cholesterol levels, insulin secretion, and diet-induced changes in hepatic mRNA and miRNA expression (Keller et al., 2018; Keller et al., 2019; Smallwood et al., 2014; Svenson et al., 2012; Coffey et al., 2017; Que et al., 2021). However, since individual DO hybrids are non-reproducible, each mouse needs to be independently genotyped, and it is not possible to make intra-strain comparisons across multiple studies.

### Um-Het3 (Het3)

The Het3 is a heterozygous mouse population first generated by the Core Facility for Aged Rodents (CFAR) program and has since been utilised by the National Institute on Ageing (NIA) Interventions Testing Program (ITP), which aims to identify non-invasive interventions that extend lifespan and promote healthy ageing (Nadon et al., 2008). The breeding scheme involves a four-way cross between (BALB/cJ × C57BL6/J) F_1_ females with (C3H/HeJ × DBA/2J) F_1_ males (Miller and Chrisp, 1999). Therefore, the F_2_ offspring are derived from inbred grandparents with known linkage phase and have relatively large allelic variation. Furthermore, studies conducted by the ITP have been performed at three separate experimental sites under strict environmental conditions to account for site-to-site variations (Nadon et al., 2008). The main objective of studies using the Het3 population has been to identify interventions with robust effects across a range of genetic backgrounds, though this panel has also been used to map QTLs for various traits (Jackson et al., 1999; Bou Sleiman et al., 2022). Het3 mice have mostly been utilised for the identification of potential anti-ageing compounds, several of which (e.g. metformin, rapamycin, acarbose, canagliflozin) were reported to have a favourable impact on metabolic profile and other cardiometabolic-related traits, though often in a sex-dependent manner (Zhu et al., 2022; Miller et al., 2014; Herrera et al., 2020; Miller et al., 2020; Snyder et al., 2023). Other studies have used Het3 mice to study the influence of diet on cardiometabolic-related traits (Miller et al., 2014; Zheng et al., 2022; Green et al., 2022). Finally, since Het3 mice are genetically unique, genetic and phenotypic data can be pooled across studies to generate a powerful, cumulative resource for the genetic dissection of traits (Bou Sleiman et al., 2022).

### Hybrid Mouse Diversity Panel (HMDP)

**Figure 1.**
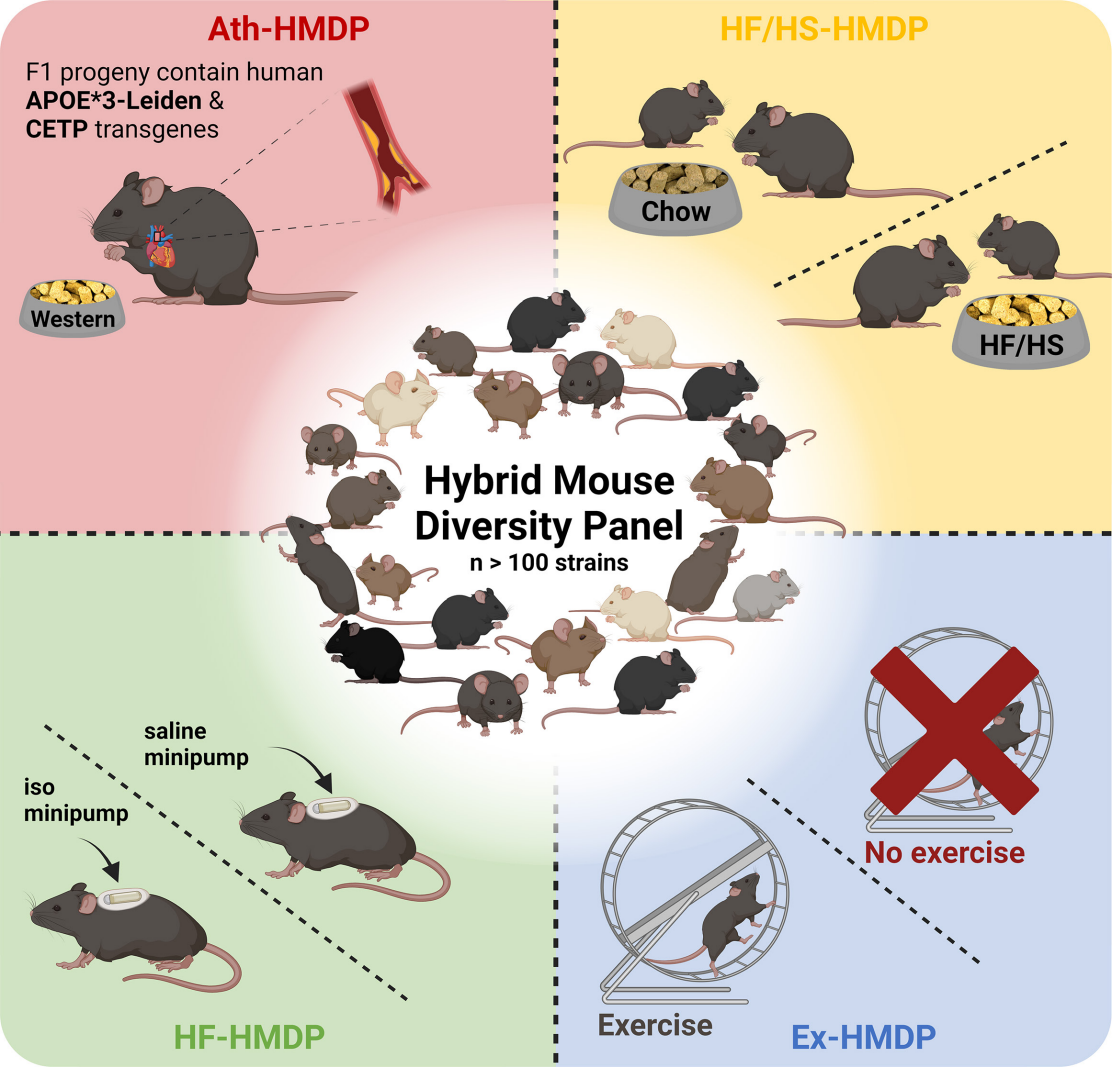
Illustrative overview of Hybrid Mouse Diversity Panel (HMDP) study designs utilised for the investigation of cardiometabolic diseases and related traits. Interventions include transgenic expression of the human apolipoprotein (APO)E*3-Leiden and the human cholesteryl ester transferase protein (*CETP*) transgenes with concomitant feeding of an atherosclerosis promoting western diet (Ath-HMDP; red) (Bennett et al., 2015), feeding of a high-fat, high-sucrose diet (HF/HS-HMDP; yellow) (Parks et al., 2013), induction of isoproterenol (iso)-induced HF (HF-HMDP; green) (Rau et al., 2015), and 30 days of voluntary wheel running (Ex-HMDP; blue) (Moore et al., 2019). *Created with* BioRender.com.

The HMDP consists of 100+ genetically diverse inbred strains of mice that have undergone extensive genotyping (Bennett et al., 2010; Yang et al., 2009; Ghazalpour et al., 2012). The panel consists of ~30 classical and 70+ recombinant inbred strains, including many BXD strains, to enhance mapping power (Lusis et al., 2016). Although mapping resolution is inferior to outbred designs such as the DO, the HMDP strains are also sufficiently powered to map highly complex traits (Ghazalpour et al., 2012). One consideration in mapping complex traits using hybrid lines derived from common ancestors lies in stratification of loci, where spurious genetic associations can, on occasion, be interpreted as causal due to genetic relatedness. Fortunately, application of linear mixed models can mostly account for this kinship (often referred to as population structure) and reduce these influences (Sul et al., 2018). The inbred design comes with the advantage of leveraging strain replicates from association mapping, which means that substantially less mice are required to achieve the same level of significance. Importantly, the HMDP is sufficiently powered to detect loci with small effect sizes for a given trait (Bennett et al., 2010). This is particularly useful for studying cardiometabolic traits, which are largely polygenic. Like other inbred mouse panels, data can be compared across generations and between cohorts (Hui et al., 2018). The HMDP was first studied in 2010, to perform fine mapping of plasma lipids (Bennett et al., 2010) and has since been used to identify novel loci for blood (Davis et al., 2013; Zhou et al., 2015), bone (Calabrese et al., 2012; Farber et al., 2011; Hiyari et al., 2015), inflammatory (Pirie et al., 2019; Orozco et al., 2012; Hiyari et al., 2015; Buscher et al., 2017), auditory (Boussaty et al., 2020; Lavinsky et al., 2015; Lavinsky et al., 2016) and behavioural traits (Park et al., 2011) in addition to cardiometabolic diseases and related traits, which we will focus on in this review (Figure 1).

The HMDP has been studied in a basal setting and following genetic, pharmacological, and lifestyle interventions, to mimic features of cardiometabolic diseases or healthy interventions as described below. For example, to explore the genetic regulation of the hepatic and plasma lipidome, Parker and colleagues integrated genetic data with liver proteomics and lipidomics, as well as plasma lipidomics from 107 HMDP strains, in the absence of intervention (Parker et al., 2019). Through a series of bioinformatic analyses, this resource was leveraged to capture both known and novel targets with therapeutic potential for the regulation of lipid metabolism, of particular relevance to settings of lipid dysregulation such as fatty liver disease.

Atherosclerosis phenotypes have also been studied using the HMDP. In this body of work, male C57BL/6J mice with humanised lipid profiles, via transgenic expression of the human cholesteryl ester transferase protein (*CETP*) transgene (Jiang et al., 1992) and the human apolipoprotein (APO)E*3-Leiden variant (van den Maagdenberg et al., 1993), were interbred with female HMDP mice to generate a panel of >100 genetically diverse F_1_ hybrid atherosclerosis-prone mice. Hybrid offspring were subsequently fed a high-fat, high-cholesterol (western) diet for 16 weeks (Ath-HMDP; Figure 1; Bennett et al., 2015). These studies identified many novel genes mapping to plaque burden and atherosclerosis-related traits, and have been used as a complementary resource to omics data generated from human tissues to identify evolutionarily conserved pathways that drive atherosclerosis, and prioritise high confidence candidates for further investigation (Talukdar et al., 2016; Koplev et al., 2022; von Scheidt et al., 2017; Cohain et al., 2021; Kessler et al., 2017; Bauer et al., 2022; von Scheidt et al., 2021; Li et al., 2022).

To explore the genetic architecture of HF, Rau and colleagues treated mice from 105 strains of the HMDP with isoproterenol for 21 days to mimic features of heart failure (HF-HMDP; Figure 1; Rau et al., 2015). Further studies have leveraged this platform to provide insights into the genetic regulation of traits related to cardiac dysfunction including cardiac remodelling (Wang et al., 2016), hypertrophy (Santolini et al., 2018; Rau et al., 2017; Krishnan et al., 2022), diastolic dysfunction (Wang et al., 2019; Cao et al., 2022) and altered cardiomyocyte metabolism (Seldin et al., 2017), and further facilitated the identification of transmembrane glycoprotein NMB (GPNMB) as a potential plasma biomarker for left ventricular mass (Lin et al., 2018).

HMDP mice have also been subjected to lifestyle interventions such as an 8-week HF/HS diet (HF/HS-HMDP; Figure 1), alongside chow-fed control mice, to explore the genetic drivers of diet-induced obesity and gut microbiota (Parks et al., 2013). Subsequent studies have used this platform to elucidate genetic mechanisms that regulate hepatic triglyceride (TG) content (Hui et al., 2015), hepatic lipids ([Bibr bib149][Bibr bib147]), sex- and tissue-specific mechanisms of NAFLD (Chella Krishnan et al., 2018; Norheim et al., 2021; Parks et al., 2015; Kurt et al., 2018; Norheim et al., 2017; Chella Krishnan et al., 2021a) and phenotypes linked to adiposity, mitochondrial function, and insulin resistance (Civelek et al., 2017; Chella Krishnan et al., 2019; Chella Krishnan et al., 2021b; Schugar et al., 2017).

Finally, the HMDP has been utilised to provide novel insights into the genetic and molecular mechanisms underpinning protective metabolic interventions. For example, a 15-strain subset of the HMDP was subjected to 30 days of voluntary wheel-running (Ex-HMDP; Figure 1) to identify novel mechanisms that regulate mitochondrial biogenesis and bioenergetics in skeletal muscle (Moore et al., 2019). Further analysis of metabolic tissues from these mice could provide valuable insight into the mechanisms that underlie the complex biological response to exercise, which could be leveraged to identify novel therapeutic targets for the treatment of cardiometabolic diseases.

### Integration of human and mouse data for biological discovery

The development of GRPs in model organisms such as those described above, which have largely conserved genetics, has proven to be a valuable resource for the identification of genes associated with complex phenotypes. However, their utility is somewhat confounded by their as yet, mostly unproven translation to human disease. To this end, the integration of human and mouse genetic data can provide a novel opportunity for target identification and functional annotation that cannot be achieved by either method alone. The large sample size of human GWAS provide considerable statistical power, whereas mouse systems offer greater environmental control and provide an opportunity to perform detailed phenotyping in relevant disease settings, as well as access to pertinent tissue samples. When combined, these resources provide an enhanced ability to identify susceptible loci with human disease relevance and elucidate underlying molecular mechanisms. Importantly, emerging studies have demonstrated that the genes and gene regulatory networks that modulate biological processes in mice are largely conserved in humans (Li et al., 2020; Talukdar et al., 2016; Koplev et al., 2022; von Scheidt et al., 2017; Benegiamo et al., 2023). This cross-species concordance demonstrates that many of the genetic mechanisms that underlie human disease can indeed be accurately identified using mouse GRPs, confirming that integration of mouse and human data is advantageous in this context.

**Figure 2.**
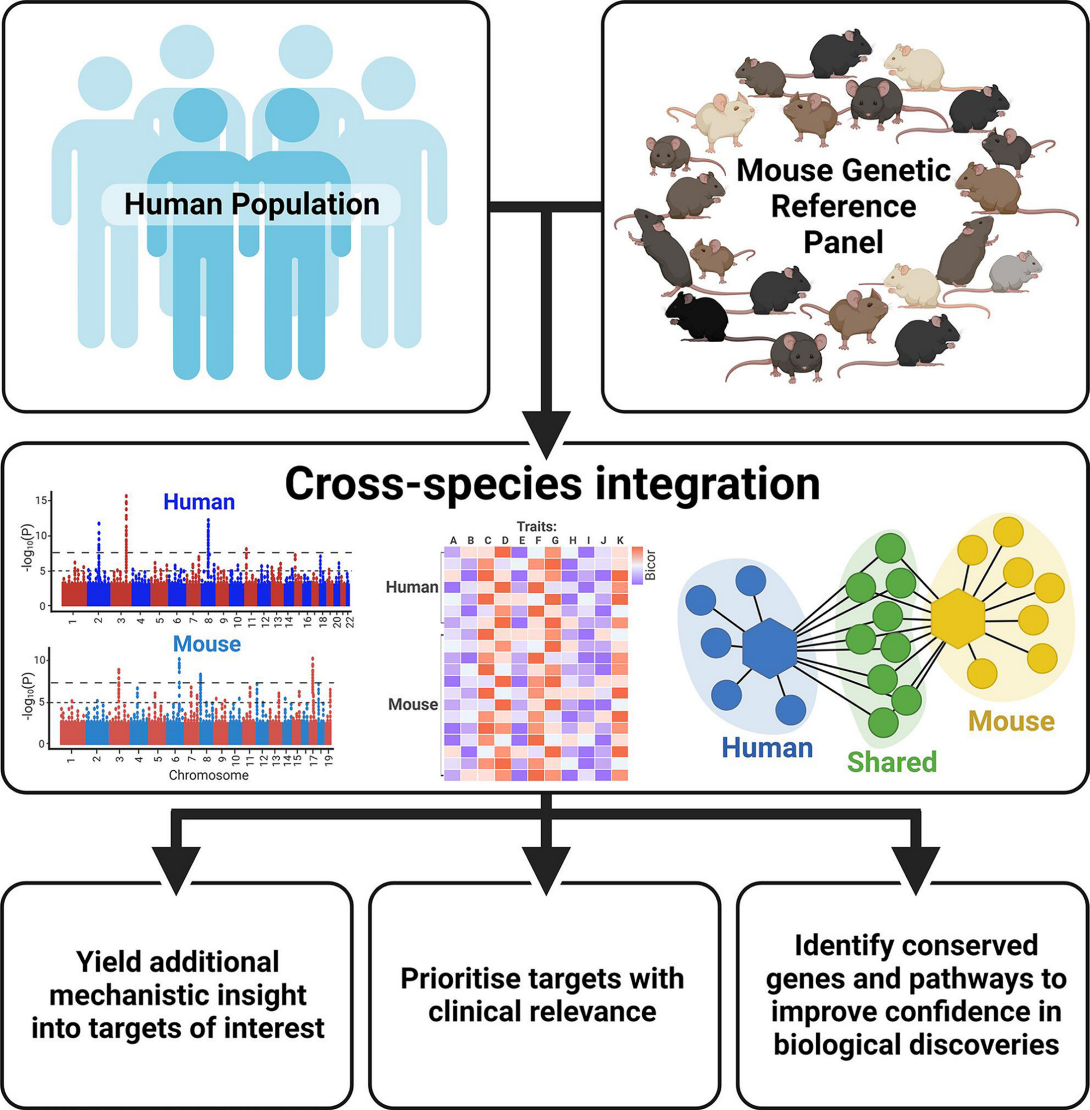
Benefits of integrating human and mouse datasets for biological discovery. Created with BioRender.com.

With thousands of genetic loci having been identified in human GWAS, research has shifted focus in the post-GWAS era from discovery of novel genetic variants to downstream elucidation of biological function. Indeed, genomic tools and experimental approaches can provide functional insight into genotype-phenotype associations (Gallagher and Chen-Plotkin, 2018). However, the discovery rate of susceptible loci exceeds the capacity to perform the time-consuming validation experiments (Stacey et al., 2019). Integrating complementary human and mouse resources can further help to pinpoint the potential causal genes and offer novel insight into how they cause disease, allowing the most promising targets to be prioritised for downstream analysis (Figure 2).

It is worth noting that there are some obvious challenges in integrating human and mouse genetic data. With regard to genetic architecture, mice have 19 pairs of autosomal chromosomes, compared with 22 in humans. Despite these obvious structural differences, it is clear that many features remain consistent between genomes, albeit they have been rearranged in large, conserved regions, known as synteny blocks, reflecting divergence of human and mouse lineages ~75 million years ago (Nadeau and Taylor, 1984; Waterston et al., 2002). Human and mouse genomes each contain ~30,000 protein-coding genes (Waterston et al., 2002). Approximately 80% of mouse genes can be mapped to a single orthologous gene (i.e. 1:1 orthologues) within the human genome and >99% map to one or more orthologous genes (Waterston et al., 2002). Hence, ~19% of mouse genes contain more than one orthologue due to lineage-specific duplication events. Such is the case for ApoE, of which three isoforms (ApoE2, ApoE3, and ApoE4) exist in humans, while only a single ApoE isoform exists in mice (Getz and Reardon, 2016). The resulting isoforms are structurally and functionally distinct and thus have different effects on lipoprotein metabolism, and indeed disease (Davignon et al., 1999). A major challenge that remains is identifying the proportion of genes that are functional orthologues (i.e. those that translate into a protein with a similar structure and biological function). Furthermore, the relative contribution of a gene, gene network, or molecular pathway to a disease will often vary with respect to disease severity. Thus, an important consideration is the use of appropriate preclinical disease models that exhibit clinically relevant features with appropriate severity. A major challenge here is that it is often difficult to accurately quantify the progression of complex diseases, where multiple insults and indeed molecular pathways can contribute to disease pathology. Such is the case for NAFLD/NASH, where the current paradigm suggests that multiple ‘hits’ (i.e. insulin resistance, hepatic lipid accumulation, inflammation, etc.) are required for disease progression in genetically predisposed individuals (Buzzetti et al., 2016). While liver biopsy or MRI provide a surrogate measure of NAFLD/NASH, bona fide, reliable biomarkers are still mostly lacking, leading to inconsistent phenotyping between individuals and indeed across different experimental settings, hampering cross-species integration.

Numerous efforts have been made to integrate datasets obtained from human and mouse populations to elucidate drivers of cardiometabolic diseases (Table 3). These include studies that have mined publicly available human or mouse databases to derive additional insight into specific targets (Parker et al., 2019; Kessler et al., 2017; Lin et al., 2018). A handful of recent studies have performed more extensive integration of genetic and multi-omics data from human and mouse populations (von Scheidt et al., 2017; Hui et al., 2018; Bauer et al., 2022; von Scheidt et al., 2021), yielding important insights that were not able to be obtained through either method in isolation, as discussed in more detail in the following section. Developing a comprehensive understanding of the genetic architecture of cardiometabolic diseases is fundamental to the selection of candidate therapeutic targets. This is underscored by recent evidence indicating that selecting therapeutic targets in humans that have evidence of genetic linkage with the disease under investigation more than doubles the success rate of drugs that enter the clinical pipeline (Nelson et al., 2015; King et al., 2019). Clinical translation has likely been hampered by the fact that preclinical studies are often performed in only a single strain of mice, commonly C57BL/6J in the context of metabolic studies, which is not reflective of the genetic and phenotypic heterogeneity of a human population. For example, while most strains (including C57BL/6J) exhibit metabolic dysfunction in response to high-fat diet feeding, albeit with varying magnitude, certain strains are protected from diet-induced insulin resistance (i.e. BALB/c, WSB/EiJ and CAST/EiJ), hepatic fibrosis (i.e. CAST/EiJ), or weight gain (i.e. A/J, WSB/EiJ and CAST/EiJ), while others (i.e. PWK/PhJ) are more susceptible (Montgomery et al., 2013; Bachmann et al., 2022; Benegiamo et al., 2023). Furthermore, an increase in energy expenditure was observed in response to a protein-restricted diet in male and female C57BL/6J mice, but not in DBA/2J or Het3 mice (Green et al., 2022). These findings highlight the value of engaging GRPs with substantial genetic diversity for therapeutic discovery and validation, as they test targeted responses to a given intervention in multiple diverse strains, as opposed to the response in just one genetic background. Thus, when deciding on strain selection for a given discovery or validation study, especially when the trait under investigation has a polygenic architecture, it may be beneficial to include multiple strains, within logistical constraints, with as much genetic and relevant phenotypic diversity as possible.

**Table 3.** Select examples of studies that have incorporated human and mouse data for biological discovery.

Cross-species integration	Trait/description	Cross-species conserved QTL(s), Gene(s), PROTEIN(S), or networks with trait of interest	Experimentally validated Gene(s)/PROTEIN(S)	*Reference
***Human-to-mouse integration***	Atherosclerosis/CAD	*PVRL2* (*NECTIN*-2/*CD112*)	–	Bennett et al., 2015
	Atherosclerosis/CAD	12 gene networks	*AIP*, *DRAP1*, *POLR2I*, *PQBP1*	Talukdar et al., 2016
	Atherosclerosis/CAD and plasma lipids	66 genes in aorta and 27 in liver for atherosclerosis 151 genes in liver for plasma lipids	–	von Scheidt et al., 2017
	Biomarker for atherosclerosis/CAD	*GUCY1A3*	*GUCY1A3*	Kessler et al., 2017
	Glucose and lipids in atherosclerosis/CAD	Glucose and lipid determining gene network	*LSS*	Cohain et al., 2021
	Atherosclerosis/CAD and cholesterol liver networks	*MAFF*	*MAFF*	von Scheidt et al., 2021
	Cross-tissue endocrine factors regulating CAD gene networks	42 endocrine factors	*EPDR1*, *FCN2*, *FSTL3, LBP*	Koplev et al., 2022
	Atherosclerosis/CAD	55 genes conserved for atherosclerosis; 14 conserved for other cardiovascular-related traits	*RGS19, KPTN*	Li et al., 2022
	Atherosclerosis/CAD and cholesterol liver networks	Liver subnetwork consisting of 50 genes, including the key driver gene, *ATF3*	*ATF3*	Bauer et al., 2022
***Mouse-to-human integration***	Blood pressure	*Ubp1*	–	Koutnikova et al., 2009
	Diabetes-related traits	Syntenic regions identified for 49 QTLs for gene modules and physiological traits	–	Keller et al., 2018
	Cross-tissue endocrine interactions regulating whole-body metabolism	*Lcn5*/*LCN6*, *Notum*	*Lcn5*/*LCN6*, *Notum*, SMOC1, ITIH5, PPBP	Seldin et al., 2018
	Biomarker for heart failure	GPNMB	–	Lin et al., 2018
	Hepatic fibrosis	Nine conserved pathways	–	Hui et al., 2018
	Hepatic and plasma lipidome	PSMD9	*Psmd9*	Parker et al., 2019
	Exercise metabolism	*Dnm1l*	*Dnm1l*	Moore et al., 2019
	Diabetes-related traits	*Hunk*, *Zfp148* (others not reported)	*Ptpn18*, *Hunk*, *Zfp148*	Keller et al., 2019
	Cholesterol metabolism	54 genes	*Sesn1*	Li et al., 2020
	NASH/NAFLD	L-PK (*Pklr*)	L-PK (*Pklr*)	Chella Krishnan et al., 2021a
	NASH	Up to 42% or 35% overlap of upregulated or downregulated genes in NASH, depending on mouse strain	–	Benegiamo et al., 2023

* For brevity, a selection of key references are provided in this table. We apologise to the investigators whose work could not be cited due to space limitations.

NAFLD, non-alcoholic fatty liver disease; NASH, non-alcoholic steatohepatitis; CAD, coronary artery disease.

### Leveraging human-to-mouse integration for biological validation and mechanistic insight

Loci identified in human GWAS can contain many genetic variants in linkage disequilibrium and it can therefore be challenging to pinpoint the causal SNP that is responsible for variation of a given phenotype. Overlaying gene expression data from human samples can facilitate the identification of candidate genes by identifying potential *cis*-eQTLs that co-map with a trait of interest. However, there is often limited access to the relevant human tissues, and the diverse conditions under which human tissue is collected can influence the quality of downstream eQTL data (Votava and Parks, 2021). These limitations can be circumvented by combining human GWAS loci with genetic mapping data obtained from mouse GRPs. For example, SNPs such as rs2075650 near *APOE* and *APOC1* in humans, genes that have well established roles in lipid metabolism and atherosclerosis, have been associated with CAD risk (Deloukas et al., 2013). A significant correlation between aortic root lesion area and the expression of poliovirus receptor related 2 (*Pvrl2,* also known as *Nectin-2* or C*d112*) was identified across the Ath-HMDP in both the aorta and liver. This gene is found in the same locus as *APOE* and *APOC1* and was speculated to partly mediate the effect of rs2075650 (Bennett et al., 2015). Thus, cross-species interrogation can be useful for pinpointing candidate genes that contribute to polygenic diseases.

Polygenic diseases are often influenced by the interaction of gene networks within and between multiple tissues. Network modelling approaches can assist in elucidating the genetic circuits within one or multiple tissues that drive biological or pathological processes. One such approach is weighted gene co-expression network analysis (WGCNA), a global expression analysis tool developed by Zhang and Horvath, 2005, that aims to identify modules of co-expressed genes. Modules can subsequently be tested for association with a trait of interest, thereby allowing annotation of gene networks that are enriched for a given phenotype or in a disease setting (Chen et al., 2008; Lusis et al., 2008). Using this approach, Talukdar and colleagues integrated gene expression from seven tissues obtained from individuals with late-stage CAD from the Stockholm Atherosclerosis Gene Expression (STAGE) cohort, to construct tissue-specific and cross-tissue co-expression modules for CAD phenotypes (Talukdar et al., 2016). This led to the identification of 30 ‘CAD-causal’ modules consisting of genes which contained either a *cis*-eQTL or had previously been reported in human CAD GWAS. Integration with phenomic and multi-tissue global gene expression data from 105 strains of mice from the Ath-HMDP revealed that 46% of the CAD-causal modules were associated with phenotypes related to atherosclerosis in mice. Among these, they identified one tissue-specific regulated gene network in aorta, containing 109 genes, that was enriched 2.5-fold in association with lesion size across the Ath-HMDP. Using the key driver analysis (KDA) algorithm, this network was shown to be regulated by seven key driver genes, or ‘hub genes’. Such genes are central to the regulation of entire gene networks and thus would be obvious candidates for therapeutic intervention due to the widespread impact that would likely result from their manipulation. Indeed, independent small interfering RNA knockdown of four key driver genes activated other genes within the network and modulated THP-1 foam cell formation, validating their involvement in the regulation of atherosclerosis.

SNPs in genes that regulate lipid and glucose pathways can contribute to atherosclerosis susceptibility (Björkegren and Lusis, 2022). To explore gene regulatory networks related to lipid and glucose traits in CAD, Cohain and colleagues performed differential expression analysis followed by co-expression analysis on gene expression data from seven tissues from the STARNET study (Cohain et al., 2021). One such module, termed the glucose and lipid determining module (GLD), was negatively associated with cholesterol traits and positively associated with glucose traits. Furthermore, the association between the GLD co-expression module and glucose and lipid traits was replicated in two independent human cohorts and in mice from the HMDP and the Ath-HMDP. Four key driver genes were identified, all of which are known regulators of cholesterol biosynthesis. Notably, the ability to reconstruct the GLD module across several independent human and mouse cohorts provides a clear indication that this module and the four predicted key drivers are evolutionarily conserved mediators of glucose and cholesterol metabolism.

In a separate study, Chella Krishnan and colleagues demonstrated that adipose mitochondrial oxidative phosphorylation (OXPHOS) genes were elevated in female mice across the HF/HS-HMDP in a tissue-specific and sexually dimorphic manner. This effect was conserved across the human STARNET and GTEx cohorts (Chella Krishnan et al., 2021b). An association was also observed between mitochondrial DNA levels and metabolic traits such as body weight and insulin resistance across the HF/HS-HMDP that was conserved across the human METSIM cohort. Association mapping of the HF/HS-HMDP led to the identification of a female-specific *trans*-eQTL regulated by NADH:ubiquinone oxidoreductase core subunit V2 (*Ndufv2*) that controlled the expression of 89 genes, of which approximately one third were OXPHOS genes. Subsequent validation experiments confirmed a causal role for *Ndufv2* in the sex-specific regulation of obesity via alterations in mitochondrial function.

Mouse and human data can also be integrated by mapping synteny blocks between genomes. For example, in ~500 HF/HS-fed DO mice, Keller and colleagues identified genetic variants in pancreatic islets associated with diabetes-related traits *in vivo*, including plasma insulin and glucose levels following fasting, or in response to an oral glucose tolerance test (Keller et al., 2018), and in cultured islets treated *ex vivo* with insulin secretagogues (Keller et al., 2019), as previously mentioned. In each study, QTL mapping was performed for *in vivo* and *ex vivo* traits and syntenic regions in mice were mapped to the human genome and compared with human SNPs related to type 1 diabetes and T2D to identify conserved diabetes-associated variants. The authors observed that syntenic regions containing mouse *in vivo* and *ex vivo* QTL were significantly enriched with diabetes-related SNPs in humans, with over half of the *ex vivo* QTL mapping to one or more diabetes-related traits in humans.

### Leveraging mouse-to-human integration for improved clinical relevance and translation

Human datasets can be used to validate findings from mouse discovery datasets. For example, using the HMDP, proteasome 26S subunit, non-ATPase 9 (PSMD9) was identified and experimentally validated as a novel regulator of hepatic and plasma TG and diglyceride (DG) abundance (Parker et al., 2019). These findings were complemented by analysis using S-PrediXcan (Barbeira et al., 2018), in which hepatic *PSMD9* expression was associated with several readouts of adiposity in the UK Biobank. Further supporting this, a small GWAS linked *PSMD9* to obesity and T2D (Gragnoli and Cronsell, 2007; Gragnoli, 2010; Gragnoli, 2013). These studies thus provided support for PSMD9 as a bona fide regulator of lipid metabolism in mice and humans and suggested that this protein could be a potential target of interest for clinical obesity and hepatosteatosis. Similarly, Lin and colleagues performed a mouse discovery analysis in the setting of HF, in isoproterenol-treated mice from 91 strains of the HF-HMDP (Lin et al., 2018). They complemented their findings by overlaying human cardiac transcriptomic data, which demonstrated that *Gpnmb* was significantly upregulated in the setting of HF in the MAGNet consortium, similar to the effect observed in HF-HMDP mice. Furthermore, cardiac GPNMB protein expression was increased in two separate murine models of HF, whilst plasma GPNMB abundance was reciprocally attenuated in the setting of HF in humans and mice, suggesting that plasma GPNMB may possess diagnostic or prognostic utility as a biomarker for HF.

The above studies performed ‘look-up’ type integration, with specific outcomes being tested for association. However, more non-directed approaches may provide broader utility in identifying novel, evolutionarily conserved drivers of complex disease. Indeed, in a recent study, Li and colleagues performed WGCNA using liver expression data obtained from the HMDP and DO mouse populations to construct mouse liver co-expression modules (Li et al., 2020). The enrichment of genes for the ‘cholesterol biosynthetic process’ Gene Ontology term identified a cholesterol module of 2435 genes, of which 112 consistently replicated across datasets. They next integrated the 112 genes with SNPs for plasma total cholesterol, low-density lipoprotein (LDL)-cholesterol, high-density lipoprotein (HDL)-cholesterol, and TG levels from the Global Lipid Genetics Consortium human GWAS, which prioritised a subset of 48 autosomal genes, 25 of which were novel, that were below the genome-wide and sub-threshold significance. Of the 25 prioritised genes, many corresponding loci were replicated in association with plasma lipid traits across the Million Veteran Program (MVP) and UK Biobank human lipid GWAS datasets.

### Non-directed integration of human and mouse data: moving beyond GWAS

With regard to non-directed integration of data obtained from human and mouse populations, von Scheidt and colleagues comprehensively explored the literature and catalogued all the genes from experimental mouse models that significantly altered lesion size or plaque stability (827 genes from over 9000 publications) and compared them with orthologous human GWAS-derived genes for CAD (244 genes from 169 significant and suggestive GWAS loci). They demonstrated that only 46 orthologous GWAS-derived genes had been studied in mice, 45 of which significantly impacted atherosclerosis (von Scheidt et al., 2017). Furthermore, pathway enrichment analysis for gene sets in the human GWAS and the gene set of 827 mouse atherosclerosis genes revealed that over 50% of the enriched CAD pathways were concordant between mice and humans. The authors also examined the variation in lesion size across the Ath-HMDP and found that, of the 244 human GWAS-derived CAD genes, the expression of 66 (27%) orthologous genes in the aorta and 27 (11%) genes in the liver correlated with lesion size. It is noteworthy that this may indeed underestimate the cross-species concordance by ignoring the contribution, albeit presumably minor, of extra-aortic and extra-hepatic genes that potentially associate with lesion size. By overlaying two human lipid GWAS (274 genes) with plasma lipids from the Ath-HMDP they demonstrated that the expression of 151 of 274 (55%) mouse orthologous genes in liver and adipose tissue correlated with plasma lipids across strains. This study demonstrates that there is substantial conservation of genetic mechanisms and biological pathways that influence atherosclerosis in mice and humans and provides a rich resource of cross-species conserved genes for further analysis. For example, in follow-up studies, the 244 human GWAS-derived CAD genes and 827 mouse atherosclerosis genes were mapped to Bayesian networks constructed using human and mouse genetic and gene expression data (Bauer et al., 2022; von Scheidt et al., 2021). Through investigating gene regulatory networks in atherosclerosis, these studies predicted *MAFF*/*Maff* and *ATF3*/*Atf3* to orchestrate a liver network enriched in genes linked to atherosclerosis. Bioinformatic analysis of liver gene expression data from STARNET and multiple HMDP cohorts indicated a potential role for *MAFF*/*Maff* and *ATF3*/*Atf3* in the context-specific regulation of lipid and lipoprotein metabolism via the low-density lipoprotein receptor (*LDLR*/*Ldlr*). These findings were validated *in silico* and *in vitro*, suggesting that *MAFF*/*Maff* and *ATF3*/*Atf3* regulate lipid metabolism and atherosclerosis in an inflammation-dependent manner.

Hui and colleagues combined GWAS analysis with liver transcriptomic data from 102 strains of the Ath-HMDP to identify genetic loci that contribute to hepatic fibrosis, a key feature of NASH (Hui et al., 2018). This led to the identification of several *cis*-eQTLs, including those for phosphatase and actin regulator 2 (*Phactr2*) and eukaryotic translation initiation factor 3 subunit H (*Eif3h*), the gene expression of which significantly correlated with liver fibrosis. In subsequent analyses, mouse hepatic gene expression data was compared to gene expression in liver explants of 68 individuals undergoing bariatric surgery, including a subset of individuals with histologically diagnosed NASH (León-Mimila et al., 2015). Using marker set enrichment analysis to identify GWAS signals that were overrepresented among eQTLs mapped to individual pathways, a 60% overlap between mouse and human NASH sub-networks was identified. Specifically, they identified nine pathways that were shared between mouse and human (e.g. innate and adaptive immune system), in addition to six mouse-specific (e.g. fatty acid, TG, and ketone body metabolism) and twelve human-specific (e.g. cytokine signalling) pathways. Collectively, these studies provide evidence that combining data obtained from mouse GRPs with data obtained from humans can assist in elucidating conserved disease-associated pathways, which have practical implications for downstream analyses. Although we are yet to see the full potential of this approach, these efforts have provided unique insight into the conservation of genes and molecular processes that confer disease susceptibility.

### Validation studies in systems genetics research

An important aspect of systems genetics is the validation of candidate targets to establish causality. Indeed, statistical causal inference techniques such as *cis*-expression correlation or mediation analysis are useful in prioritising the gene(s) that are likely to be causally associated with a given SNP. However, if phenotypic components themselves are tightly correlated, as is often the case for functionally related genes, proteins, and lipid species, this can inflate mediation statistics and increase the rate of false positives. Despite advances in statistical network modelling approaches such as WGCNA or Bayesian networking and its derivative KDA algorithm, these approaches are prone to false positive relationships due to inherent limitations in the modelling of complex and dynamic biological systems, or simply the result of bystander genes. Integrative approaches that combine the advantages of both human and mouse datasets to assign priority to the most promising candidate genes can help reduce the rate of false positive associations. However, this does not obviate the requirement for validation experiments to establish causality and confirm directionality. Further mechanistic insights can also be obtained either in the absence of tissue crosstalk using *in vitro* experimentation, or in the presence of multi-cellular and multi-organ crosstalk using *in vivo* studies, which are more representative of a biological system.

For example, upon identifying an association between PSMD9 and acylglycerol species in liver and plasma across HMDP mice, Parker et al. utilised loss- and gain-of-function experiments to further elucidate the role of PSMD9 in the context of lipid regulation *in vivo* (Parker et al., 2019). In two distinct strains of mice, C57BL/6J and DBA/2J, adenoviral overexpression of PSMD9 for 5–7 days promoted the accrual of DG and TG species in mice fed a chow diet. Strikingly, in mice fed a western diet for 28 days to promote hepatic lipid accrual, PSMD9-antisense oligonucleotide treatment was associated with a significant attenuation of hepatic and plasma TG and DG levels in DBA/2J mice, and to a lesser extent in C57BL/6J mice. Indeed, the phenotypic outcome resulting from modulation of a gene is highly dependent on genetic background, as others have previously demonstrated (Sittig et al., 2016). This strain-specific effect is representative of the variable influence that modulation of a single gene would have on a complex trait in a human population and underscores the importance of performing experimental validation in multiple strains with differing genetic backgrounds, where possible.

Cardiometabolic diseases are greatly influenced by biological sex. This highlights the importance of incorporating both sexes in validation experiments, where possible, in line with efforts to promote inclusion of females in preclinical research (Clayton and Collins, 2014; Heidari et al., 2016). Some studies have addressed this by performing validation studies in male and female mice (Moore et al., 2019; Schugar et al., 2017), while others have validated findings in ovariectomised and/or gonadectomised mice to explore how sex hormones regulate complex traits and disease (Parks et al., 2015; Chella Krishnan et al., 2021b; Norheim et al., 2019). For example, this approach revealed that sex hormones regulate key sex- and tissue-specific pathways in NAFLD (Kurt et al., 2018). Inclusion of sex as a biological variable in validation studies improves the applicability of findings and can, to an extent, facilitate personalised management strategies.

With thousands of loci having been identified for complex diseases, a remaining challenge is the prioritisation of high-confidence genes with therapeutic potential. For example, as mentioned above, Li and colleagues integrated mouse liver gene expression networks with data from a human lipid GWAS. This led to the identification of 25 novel genes implicated in cholesterol metabolism (Li et al., 2020). Using a variety of functional experiments to filter the genes, they demonstrated that hepatic expression of many of these genes was modulated in response to diets that modulate hepatic cholesterol levels, or in response to cholesterol depletion in mouse liver cells. Furthermore, silencing of 10 of the 25 genes altered the expression of the regulator of cholesterol synthesis, hydroxymethylglutaryl-CoA synthase (*Hmgcs*), in mouse liver cells. This led to the identification of several high-confidence, cross-species conserved, cholesterol regulating genes, such as *sestrin1* (*Sesn1*), which was functionally characterised to play a role in cholesterol biosynthesis using *in vivo* and *in vitro* systems. This is an elegant example of how systematic, data-driven validation studies can be used to assign priority to candidate genes and provide insight into the molecular mechanism of a gene under investigation.

### Conclusions

Although the ability of human GWAS to consistently identify the causal gene(s) within a given locus for complex traits has been impeded by numerous logistical challenges, such methodologies have pioneered the gene discovery revolution and have been the springboard to contemporary genetic analytical approaches. As a complementary approach to GWAS in humans, GRPs in model organisms such as mice have also been vastly informative. As stand-alone methods, both human and mouse GWAS have been successful in their own right at identifying causal genetic variants, however their integration stands to elevate their individual successes. We have highlighted here the many potential advantages of comparing, overlaying, and integrating complementary datasets between mice and humans to improve these outcomes. Indeed, more extensive integration of data from human and mouse populations can be leveraged to maximise biological discovery and clinical translation. However, more work is necessary to advance this knowledge towards prognostic, diagnostic, and therapeutic outcomes.

### Future considerations

As the field of systems genetics rapidly progresses, there are several important points to consider in order to harness the full potential of existing and emerging resources and maximise clinical impact. In order to comprehensively and accurately integrate datasets from human and mouse populations, a number of key areas must be addressed. Firstly, future studies must address the lack of consistency in phenotyping complex traits across species. Secondly, cross-species genetic maps need to be streamlined and require improved annotation of functional orthologues, so that more direct comparisons can be made between model systems and human biology. Thirdly, the development of user-friendly webservers with gene-, SNP-, or phenotype-lookup functions, such as GeneNetwork.org (Williams and Mulligan, 2012), Starnet.mssm.edu (Koplev et al., 2022), CoffeeProt.com (Molendijk et al., 2021) the METSIM metabolomics PheWeb (Pheweb.org/metsim-metab) (Yin et al., 2022), or Institutional Specific Portals (i.e. Baker Institute Lipidomics PheWeb; Metabolomics.baker.edu.au) (Cadby et al., 2022) would be useful for those who lack the statistical or computational expertise to analyse complex datasets, providing the broader scientific community access to such datasets and the opportunity to incorporate systems genetics data into their research. Lastly, while genomic tools and complementary human and mouse populations serve as useful sources of biological validation and mechanistic insight, these should be viewed as an accompaniment, rather than a replacement for laboratory-based experimental validation. Indeed, we emphasise the importance of performing validation experiments in both sexes and across multiple genetic backgrounds, where possible, to improve the biological relevance and translation of findings. Addressing these factors will facilitate accurate and comprehensive integration of multi-omics datasets across species and enhance biological discovery and importantly, clinical translation.

With the systems genetics field now at a major crossroads on how to best leverage contemporary methodologies including PGS and Mendelian randomisation, several questions remain that will ultimately influence the direction taken in years to come. The full potential of cross-species data integration remains to be seen, with the field continuing to evolve with regard to greater depth of sample analyses, larger cohort numbers, and high-throughput analytical approaches being developed. So, what is needed from existing infrastructure to convince both clinical and discovery scientists that genetic information is a powerful complementary tool to guide discovery and therapeutic translation? Do we need to integrate genetic information from multiple ethnicities, in larger numbers and continue to push the boundaries in computational science? Although identifying disease-associated variants is undoubtedly important, should we place more emphasis on overlaying multiple biological layers such as the abundance of genes, proteins, and other intermediate metabolites to improve our confidence in the most likely causal gene(s) and underlying biology? What does full integration of human and mouse data look like and how far should this be pursued? Can we perform cross-species meta-analysis to generate more precise and powerful comparisons? Can we develop an approach to comprehensively define functional orthology across species to facilitate biological interpretation between humans and mice? Whatever the answers may be, the field must work through these challenges in a systematic and collaborative manner. Undoubtedly, these approaches, combined with addressing the abovementioned limitations, are likely to provide unique opportunities for discovery and clinical translation to improve human health and pave the way for a new direction for the field of systems genetics, greatly facilitating progress towards precision medicine.
